# Sexual Function in Levothyroxine-Treated Hypothyroid Women and Women without Hypothyroidism: A Case-Control

**DOI:** 10.3390/ijerph17124325

**Published:** 2020-06-17

**Authors:** Benjamín Romero-Gómez, Paula Guerrero-Alonso, Juan Manuel Carmona-Torres, José Alberto Laredo-Aguilera, Diana Patricia Pozuelo-Carrascosa, Ana Isabel Cobo-Cuenca

**Affiliations:** 1 Hospital El Tomillar de Sevilla, Servicio Andaluz de Salud (SAS), 41500 Alcalá de Guadaira, Spain; volvart@hotmail.com; 2 Centro de Salud Najera, Servicio Rioja Salud, 26300 Najera, Spain; pguerrero@riojasalud.es; 3 Facultad de Fisioterapia y Enfermería de Toledo, Universidad de Castilla la Mancha, 45071 Toledo, Spain; dianap.pozuelo@uclm.es (D.P.P.-C.); anaisabel.cobo@uclm.es (A.I.C.-C.); 4 Grupo de Investigación Multidisciplinar en Cuidados (IMCU), Universidad de Castilla la Mancha, 45071 Toledo, Spain; josealberto.laredo@uclm.es; 5 Instituto Maimónides de Investigación Biomédica de Córdoba (IMIBIC), 14004 Córdoba, Spain; 6 Facultad de Ciencias de la Salud, Universidad de Castilla la Mancha, Talavera de la Reina, 45600 Toledo, Spain

**Keywords:** hypothyroidism, sexual dysfunction, physiological, health, women’s, thyroid hormone

## Abstract

*Background:* Levothyroxine is the most common treatment to normalize thyroid hormones levels and to reduce primary hypothyroidism symptoms. *Aim:* To assess sexual function in women with levothyroxine-treated hypothyroidism and women without hypothyroidism. Methods: A case-control study was performed with 152 women with levothyroxine-treated hypothyroidism and 238 women without hypothyroidism. An online survey was used to collect socio-demographic data and the answers to the Women Sexual Function (WSF) questionnaire. *Results*: Women with levothyroxine-treated hypothyroidism showed a higher prevalence of sexual dysfunction than women in the control group (31.60% vs. 16.40%), furthermore the presence of hypothyroidism increased the risk of sexual dysfunction (*p* = 0.002, OR: 2.29 (1.36−3.88)). The most affected domains were ‘desire’ (*p* < 0.001), ‘arousal’ (*p* = 0.003) and ‘penetration pain’ (*p* = 0.020). In hypothyroid women, age increased the risk of sexual dysfunctions (*p* = 0.009, OR: 1.07 (1.01−1.12)), however when age was adjusted (ANCOVA) the sexual dysfunction remained in women with hypothyroidism in all domains. *Conclusions:* Hypothyroidism is associated with an increase in the prevalence of sexual dysfunction even if treated with levothyroxine and thyroid-stimulating hormone (TSH) levels are normalized. *Relevance to clinical practice:* Sexual function in hypothyroid women should be assessed before and after starting the treatment.

## 1. Introduction

Hypothyroidism is the most common thyroid disease in Spain. Valdés et al. (2017) reported a prevalence of 9.1% (CI 8.2−10.0%) in Spanish population, increasing with age and being more common in women [1]. This pathology produces a decrease in the production of thyroid hormones (T_3_ and T_4_), usually due to disorders in the gland itself (primary hypothyroidism) or due to an alteration in thyroid-stimulating hormone (TSH) secretion (secondary hypothyroidism) by the hypophysis. T_4_ and T_3_ deficiency has important effects on the organism: fatigue, weight gain, dry skin, cold intolerance, constipation, edema, myalgia, menstrual irregularities, cognitive slowing, anxiety or depression [2].

Sexuality is also affected by hypothyroidism. An increase in the prevalence of sexual dysfunction as a consequence of this pathology has also been found in both sexes [3,4,5,6,7]. In the specific case of women, hypothyroidism has been related to disorders in desire, arousal, lubrication, satisfaction and orgasm, as well as dyspareunia [5,8,9]. Different mechanisms have been proposed in order to explain the relationship between hypothyroidism and sexual dysfunction, some direct mechanism (hyperprolactinemia, alteration of other hormonal axes [10]) but also indirect mechanisms (classic symptoms of hypothyroidism [3,5], mood disorders [3,4], autoimmune processes, changes in blood circulation in the genital area [3,4]); however, the exact causes have not been precisely defined.

The usual treatment of hypothyroidism is a hormonal replacement therapy through a daily intake of sodium levothyroxine. This medicine is a synthetic form of T_4_ hormone (tetraiodothyronine). The mechanism of action of this medicine is based on the peripheral conversion of the T_4_ into the active T_3_ hormone through the action of the deiodinase enzymes [10]. The intake of the optimum dose of this medicine leads to a normalization of T_4_ and T_3_ serum levels and, secondarily, of TSH secretion by the hypophysis. Levothyroxine is a medicine with good gastrointestinal absorption and a long serum half-life (approximately 7 days). After starting the treatment, TSH levels are measured every 6 or 8 weeks. If necessary, levothyroxine doses are corrected. Once TSH levels are normalized (0.4−4 IU/mL), women have annual reviews to control the disease [10]. The normalization of TSH levels leads to the reduction in hypothyroidism symptoms, including sexual dysfunction [5,6]. However, not all the patients seem to obtain the same benefit from the treatment, and occasionally, some symptoms persist even after reaching euthyroidism [5,11,12]. Pasquali et al. (2013) found that arousal and orgasm dysfunction persisted, whereas Oppo et al. (2011) reported the presence of disorders in orgasm even with an appropriate treatment.

Despite the higher prevalence of hypothyroidism in women, there are more studies on sexual dysfunction in men than in women [5]. Although, in last years, some research has been conducted with larger samples of women with subclinical hypothyroidism [13,14], little attention has been paid to sexuality in women with primary hypothyroidism treated with levothyroxine [4,5]. Furthermore, the studies use small samples and the patients are not always euthyroid women [5,8,9,15]. In Spain, to the best of our knowledge, there are no previous studies on this topic.

Based on these reasons, the aims of this study were: (1) to assess sexual function in levothyroxine-treated hypothyroid women with normalized TSH levels and women without hypothyroidism; (2) to assess the influence of different variables on sexual dysfunction in both groups.

## 2. Materials and Methods

### 2.1. Design and Study Population

This is a multicenter study conducted from October 2018 to March 2019. The participants were 390 euthyroid women (over 18 years old) recruited among the patients of two Primary Care Health Zones in Spain. They were divided into two groups: (1) Case Group consisted of 152 women with primary hypothyroidism treated with levothyroxine; (2) Control Group consisted of 238 women without hypothyroidism.

The inclusion criteria for the case group were: (1) previous diagnosis of primary hypothyroidism, (2) at least 6 months of levothyroxine treatment, (3) normalized TSH levels (0.4−4.0 mIU/L) [10].

Exclusion criteria were: (1) diagnosis of secondary or subclinical hypothyroidism, (2) abnormal TSH levels, (3) being pregnant or lactating, (4) diagnosis of psychiatric, endocrine, gynecological or other diseases that may affect sexuality, (5) consumption of medications that may interfere with thyroid function or sexual response (methimazole/thiamazole, chemotherapy, etc.).

The inclusion criteria for the control group were: (1) absence of thyroid disorders (clinical or subclinical hypothyroidism, clinical or subclinical hyperthyroidism), (2) euthyroidism (TSH levels 0.4−4.0 mIU/L). The exclusion criteria were: (1) abnormal TSH levels, (2) being pregnant or lactating, (3) family history of clinical or subclinical hypothyroidism, (4) consumption of medications that may interfere with thyroid function or sexual response (methimazole/thiamazole, chemotherapy, etc.), (5) diagnosis of psychiatric, endocrine, gynecological or other diseases that may affect sexuality.

### 2.2. Sample Size

Sample size was calculated using Granmo software version 7.12 (Institut Municipal d´Investigación Médica, Barcelona, Spain). In a recent study, Krysiak (2016) [15] found a prevalence of sexual dysfunction of 37% in euthyroid women with Hashimoto’s thyroiditis and of 17% in control group. Based on these data, a sample of 84 women in each group is considered sufficient with an α risk of 0.05, a β risk of 0.2, a level of confidence of 95%, and a replacement rate of 10%.

### 2.3. Instruments

An online questionnaire was used with two different instruments:

(1) Questionnaire on socio-demographic variables (age, educational level, employment status, civil status, cohabitation and sexual orientation);

(2) Questionnaire on Women’s Sexual Function (WSF) of Sánchez et al. (2004) [16]. This is a self-administered questionnaire validated in Spain consisting of 14 items with a Likert scale with five options. Questions referred to the past 4 weeks. The first six items assess different aspects of sexual response. Items 7 and 8 assess aspects related to sexuality. Finally, items 9 and 10 assess sexual satisfaction. The remaining items deal with other relevant aspects of sexual activity. Score cut-points were: desire, arousal, penetration pain (severe problem (1–3), moderate problem (4–7), without problem (8–15)); sexual satisfaction (no satisfaction (1–2), moderate satisfaction (3–5), satisfactory (6–10)); lubrication, orgasm, and anticipatory anxiety (severe problem (1), moderate problem (2), without problem (3–5)); sexual initiative (absence of initiative (1), moderate initiative (2), without problem (3–5)); communication (absence of sexual communication (1), moderate sexual communication (2), without problem (3–5)). Desire, arousal, lubrication, orgasm, penetration pain, and sexual initiative domains are used to determine the existence of sexual dysfunction. The scale showed an internal consistency of 0.895–0.897 and a reliability of 0.597–0.743.

Clinical variables were collected during patients’ annual reviews Body Mass Index (BMI), TSH levels and daily levothyroxine intake.

### 2.4. Data Collection

Convenience sampling was used. First, primary healthcare professionals from both regions were contacted and invited to participate in our study. During their annual reviews, the physicians recruited among their patients those women interested in participating who met the inclusion and exclusion criteria. They all had to be fluent in Spanish in order to understand the informed consent and complete the questionnaires. After giving this informed consent, their height and weight were measured. The results of the blood tests were collected. All blood samples were taken at 8.00 a.m. after an 8-h overnight fast. Only euthyroid women (TSH 0.4−4.0 mIU/L) were selected. Finally, they received a link to the online survey. Participants were not directly contacted by the researchers in order to respect their anonymity. The questionnaire did not collect any personal data other than the variables of this study.

### 2.5. Study Variables

#### 2.5.1. Independent Variables

The independent variables included sociodemographic and clinical variables. The sociodemographic variables were: age (quantitative), educational level (categorical), employment status (categorical), civil status (categorical), cohabitation (dichotomous). The clinical variables were: sexual orientation (categorical), BMI (quantitative/categorical), presence/absence of hypothyroidism (dichotomous), TSH levels (quantitative), levothyroxine intake (categorical).

#### 2.5.2. Dependent Variables

The questionnaire on WSF scores in the following domains: desire, arousal, lubrication, orgasm, penetration pain, sexual initiative, sexual activity satisfaction and general sexual satisfaction. These variables were used both as quantitative and categorical.

### 2.6. Statistical Analysis

The statistics program IBM SPSS version 22.0 (IBM Corp, Armonk, NY, USA) was used for statistical analysis. A descriptive analysis of the variables was performed by calculating counts (*n*) and proportions (%) of the qualitative variables and means (m) and standard deviations (SD) of quantitative variables. The Kolmogorov–Smirnov test was used to test for data normality. Data were analyzed by Student *t*-test. A χ-square test was used to analyze the categorical variables, and a Fisher’s exact test was performed when frequencies were less than or equal to 5. Finally, a univariable logistic regression model and an ANCOVA adjusted for age were performed. All hypotheses contrasts were bilateral and a value of *p* < 0.05 was considered significant in all tests.

### 2.7. Ethical Considerations

This study follows the fundamental principles of the UNESCO Universal Declaration of Human Rights, the Helsinki Declaration, and Spanish Organic Law 3/2018, of December 5, on the Protection of Personal Data and Guarantee of Digital Rights, keeping it strictly confidential and not accessible to unauthorized third parties and Regulation (EU) 2016/679 of the European Parliament and Council of April 27, 2016 on Data Protection (RGPD), of the Spanish State. The study was approved by the institutional ethical committees from both Primary Care Health Zones (Comité de Ética del Complejo hospitalario de Toledo CEIC TO-273/2018 and Málaga CEIC Ma-25/2018). 

## 3. Results

### 3.1. Clinical and Sociodemographic Variables

A total of 505 women agreed to participate in the study and 390 of them met the participation criteria and completed the online questionnaires. These women were divided into two groups: Case Group (euthyroid women with primary hypothyroidism treated with levothyroxine, *n* = 152, 38.97%) and Control Group (women without hypothyroid-ism, *n* = 238, 61.03%). The mean age of the sample was 35.49 ± 9.41 with a BMI of 23.54 ± 4.09. There were 292 (74.9%) women with university studies and 285 (73.05%) women were employed. Regarding sexual relations, 349 (89.5%) women were heterosexual, 307 (78.7%) had been in a relation for at least 6 months and 232 (59.5%) were cohabitating. The mean of TSH level was 2.22 ± 0.90. Finally, regarding hypothyroidism, autoimmune etiology was the most common (88.2%) (Table 1).

Both groups were compared according to the socio-demographic and clinical variables (Table 1) using a Student *t*-test (quantitative) and a χ-square test (categorical).

### 3.2. Women’s Sexual Function (WSF) Results

Sexual dysfunction was reported by 22.3% of the total sample. The prevalence was significantly higher (*p* < 0.001) in hypothyroid women (31.6%) than in women without hypothyroidism (16.4%). Furthermore, significant differences were found in desire (*p* < 0.001), arousal (*p* = 0.002), penetration pain (*p* = 0.016), and sexual initiative (*p* < 0.001) in case group (Table 2).

Despite this prevalence of sexual dysfunction, 91.8% of the women in the total sample were satisfied with sexual activity and 80.52% were satisfied with their general sexuality (Table 2).

Logistic regression was used to determine the effect of independent variables on the prevalence of sexual dysfunction (yes/no) (Table 3).

Women in the case group showed a higher risk of sexual dysfunction (*p* = 0.002, OR: 2.29 (1.36−3.88)) than women in the control group.

Regarding age, it showed a significant influence on the prevalence of sexual dysfunction (*p* = 0.009, OR: 1.07 (1.01−1.12)) in the case group.

The rest of variables did not show any significant relationship with the prevalence of sexual dysfunction.

Finally, an ANCOVA adjusted for age was performed. The differences in the domains of desire, arousal, lubrication, orgasm, penetration pain, anticipatory anxiety, sexual initiative, sexual communication, sexual activity satisfaction and general sexual satisfaction were significant (Table 4).

## 4. Discussion

Monotherapy with levothyroxine is the recommended treatment for hypothyroidism [17]. It is a medication with good absorption and easy to take. Moreover, it has been demonstrated to be superior to other alternative therapies [10]. Its daily intake has been related to the improvement in symptoms such as growth deficits, severe fatigue, constipation, anxiety or depression [10,17], nevertheless, some symptoms seem to persist [5,11,12].

In our study, 22.3% of women reported sexual dysfunction, so that 31.60% of women with hypothyroidism and 16.40% of women without hypothyroidism presented sexual dysfunction. These data are similar to those found by Pasquali (2013) [9] in women with thyroid disorders (26.1% in hypothyroid women vs. 20.7% in controls) and Krysiak (2016) [15] in women with Hashimoto’s thyroiditis (37% in hypothyroid women vs. 17% in controls). Both authors observed a higher prevalence of sexual dysfunction in hypothyroid women despite levothyroxine treatment. Atis et al. (2010) [3] found a higher sexual dysfunction rate in hypothyroid women (56% in non-euthyroid hypothyroid women vs. 15% in controls) which could be due to the fact that their sample consisted of hypothyroid women without treatment.

In this respect, Pasquali et al. (2013) [9] and Oppo et al. (2011) [8] found an inverse correlation between hormone levels and total WSF score. Atis et al. [3] also observed a higher prevalence of sexual dysfunction if TSH > 10. Hypothyroid women in our study reported normal TSH levels which may be the reason of not finding a significant correlation between TSH levels and the prevalence of sexual dysfunction.

For the women in our sample, despite levothyroxine treatment and normalized TSH levels, hypothyroidism increased the risk of sexual dysfunction (*p* = 0.002, OR: 2.29 (1.36−3.88)). In line with other authors [4,5,15], our research showed that the presence of sexual dysfunction in different sexual domains was significantly higher in the case group despite the use of levothyroxine. As Oppo (2011) and Pasquali (2013), we found significant differences in arousal; however, we found no differences in lubrication and orgasm. Regarding other domains, the prevalence of sexual dysfunction in desire, penetration pain, and sexual initiative were significantly higher in hypothyroid women even if they are correctly treated with levothyroxine. Krysiak (2016) also reported significant differences in desire. Dysfunctions due to penetration pain may be consequence of a decrease in desire and arousal; at the same time, lower desire may lead to lower sexual initiative.

Despite the higher prevalence of moderate dysfunction of desire and arousal in the case group, both groups reported similar levels of satisfaction regarding sexual activity and general sexuality. The reason may be that, although they reported more difficulties when initiating sexual activity, subsequent performance is similar. Thus, we did not observe any significant differences in orgasm; nevertheless, on the contrary, Krysiak (2016) [15] found lower satisfaction levels in euthyroid women with treated Hashimoto’s thyroiditis.

Regarding the rest of domains, only age showed a significant effect on sexuality in women in the case group (*p* = 0.009, OR: 1.07 (1.01−1.12)). The increase in sexual dysfunction with age is well documented and has been related to the natural process of menopause [18,19,20]. Using ANCOVA to control for the age factor, mean scores in all domains remained significantly lower in hypothyroid women. Hence, it reinforces the idea that hypothyroidism may cause dysfunction in sexual response even if treated with levothyroxine, and TSH is normalized. Since our study was conducted with a larger sample than other studies [7,8,9,15], it reinforces the findings regarding differences in sexual domains.

Different studies have shown that thyroid dysfunction can exert effects on sexual function [5] and several mechanisms have been considered as a possible cause of sexual dysfunction in both sexes. Among others, researchers have studied the influence of abnormal TSH levels [8], secondary hyperprolactinemia, the symptomatology of the disease (fatigue, somnolence, mood disorders) [5], obesity and the development of cardio-vascular disease and type 2 diabetes in the metabolic syndrome [21,22]. However, the specific causes have not yet been typified.

Besides the use of levothyroxine, several options have been considered in order to treat sexual dysfunction. The combined treatment of levothyroxine/liothyronine could be more effective to reduce sexual symptoms [12], nevertheless, the use of this combined therapy is still under research. This T_3_/T_4_ combination has also been used to treat various persistent symptoms of hypothyroidism [23,24]. With regard to non-pharmacological interventions, the efficacy of psychosexual therapy in these women should be assessed.

### Strengths and Limitations

The strengths of this study are that the sample size is larger than in similar studies and includes a wide range of age groups from young to postmenopausal women. Furthermore, the online questionnaire facilitates women’s ability to provide information about their sexuality more freely.

The study is not free from limitations. Due to the design of this study, it is not possible to establish casual relationships. Besides, since it is a self-administered questionnaire, there may be some bias in the answers.

## 5. Contributions of this Study

To the best of our knowledge, this is the first research of sexuality in euthyroid hypothyroid women under levothyroxine treatment in Spain.

This study demonstrates that, despite the standard hypothyroidism treatment and normalized TSH levels, sexual dysfunctions may persist. The most affected domains are desire, arousal and penetration pain.

Sexual functioning in hypothyroid women should be assessed before and after starting the treatment and also after every adjustment in the levothyroxine dose.

Psychosexual interventions could be planned for these women if the normalization of TSH levels does not completely eliminate sexual dysfunctions.

## 6. Conclusions

Levothyroxine-treated hypothyroid women with normalized TSH levels showed a higher prevalence of sexual dysfunction than women without hypothyroidism. Women with hypothyroidism scored significantly lower than women in the control group in desire, arousal, and penetration pain. The study shows that hypothyroidism in women is associated with sexual dysfunction that is not corrected with standard levothyroxine treatment. Further research is needed to identify the underlying mechanisms and treatments for this sexual dysfunction in hypothyroid women.

## Figures and Tables

**Table 1:** Socio-demographic and clinical variables.

Variable	Case Group	Control Group	Total	Sig. (Bilateral)
	*n* = 152	*n* = 238	*n* = 390	
	**Mean** (**SD**) *	**Mean** (**SD**)	**Mean** (**SD**)	***t*-test**
**Age**	36.58 (9.96)	34.79 (8.99)	35.49 (9.52)	0.068
**BMI**	24.41 (4.59)	22.99 (3.65)	23.54 (4.09)	0.001
	*n (%)*	*n (%)*	*n (%)*	χ²
**Educational Level**				
**Primary**	15 (9.85%)	6 (2.5%)	21 (5.4%)	*p* < 0.001
**Secondary**	39 (25.65%)	38 (15.95%)	77 (19.7%)	
**University**	98 (64.5%)	194 (81.55%)	292 (74.9%)	
**Employment Status**				
**Student**	16 (10.5%)	31 (13%)	47 (12.05%)	0.001
**Employed**	101 (66.5%)	184 (77.3%)	285 (73.05%)	
**Other**	35 (23%)	23 (9.7%)	58 (14.9%)	
**Civil Status**				
**Single**	24 (15.8%)	34 (14.3%)	58 (14.9%)	0.910
**Married/Steady**	118 (77.6%)	189 (79.4%)	307 (78.7%)	
**Other**	10 (6.6%)	15 (6.3%)	25 (6.4%)	
**Sexual Orientation**				
**Lesbian**	6 (3.95%)	8 (3.35%)	14 (3.6%)	0.934
**Bisexual**	11 (7.23%)	16 (6.7%)	27 (6.9%)	
**Heterosexual**	135 (88.82%)	214 (89.95%)	349 (89.5%)	
**Cohabitation**				
**Yes**	101 (66.5%)	131 (55%)	232 (59.5%)	0.025
**No**	51 (33.5%)	107 (45%)	158 (40.5%)	
**Hypothyroidism Etiology**				
**Postsurgical**	18 (11.8%)	-	-	
**Autoimmune**	134 (88.2%)	-	-	
**Levothyroxine Intake**				
**50−75 µg/day**	79 (52%)	-	-	
**76−125 µg/day**	61 (40.1%)	-	-	
**Over 125 µg/day**	12 (7.9%)	-	-	

* Standard deviation.

**Table 2:** Sexual function scores in case and control groups.

Variable	Case Group	Control Group	Total	Sig
**Desire**				
Severe Problem	0 (0%)	3 (1.26%)	3 (0.76%))	*p* < 0.001
Moderate Problem	24 (15.79%)	10 (4.20%)	34 (8.71%)	
Without Problem	128 (84.21%)	225 (94.54%)	353 (90.53%)	
**Arousal**				
Severe Problem	1 (0.65%)	3 (1.26%)	4 (1.02%)	0.002 †
Moderate Problem	15 (9.86%)	5 (2.10%)	20 (5.12%)	
Without Problem	136 (89.49%)	230 (96.64%)	366 (93.86%)	
**Lubrication**				
Severe Problem	3 (1.97%)	3 (1.26%)	6 (1.53%)	0.171 †
Moderate Problem	14 (9.21%)	11 (4.62%)	25 (6.41%)	
Without Problem	135 (88.81%)	224 (94.12%)	359 (92.06%)	
**Orgasm**				
Severe Problem	10 (6.58%)	11 (4.62%)	21 (5.38%)	0.304
Moderate Problem	10 (6.58%)	9 (3.78%)	19 (4.87%)	
Without Problem	132 (86.84%)	218 (91.6%)	350 (89.75%)	
**Penetration Pain**				
Severe Problem	0 (0%)	1 (0.42%)	1 (0.25%)	0.016 †
Moderate Problem	8 (5.26%)	2 (0.84%)	10 (2.56%)	
Without Problem	144 (94.74%)	235 (98.74%)	379 (97.19%)	
**Anticipatory Anxiety**				
Severe Problem	3 (1.97%)	1 (0.42%)	4 (1.02%)	0.082 †
Moderate Problem	8 (5.26%)	5 (2.10%)	13 (3.33%)	
Without Problem	141 (92.77%)	232 (97.48%)	373 (95.65%)	
**Sexual Initiative**				
Absence of Initiative	30 (19.73%)	25 (10.50%)	55 (14.10%)	*p* < 0.001
Moderate Initiative	39 (25.65%)	36 (15.12%)	75 (19.23%)	
Without Problem	83 (45.35%)	177 (74.38%)	260 (66.67%)	
**Sexual Communication**				
Absence	11 (7.23%)	18 (7.56%)	29 (7.43%)	0.074
Moderate	25 (16.44%)	21 (8.82%)	46 (11.79%)	
Without Problem	116 (76.33%)	199 (83.62%)	315 (80.76%)	
**Sexual Activity Satisfaction**				
No Satisfaction	7 (4.60%)	4 (1.68%)	11 (2.82%)	0.213 †
Moderate	9 (5.92%)	12 (5.04%)	21 5.38%)	
Satisfactory	136 (89.48%)	222 (93.28%)	358 (91.8%)	
**General Sexual Satisfaction**				
No Satisfaction	8 (5.26%)	16 (6.72%)	24 (6.15%)	0.112
Moderate	27 (17.76%)	25 (10.50%)	52 (13.33%)	
Satisfactory	117 (76.98%)	197 (82.78%)	314 (80.52%)	
**Presence of Sexual Dysfunction**				
Yes	48 (31.6%)	39 (16.4%)	87 (22.3%)	*p* < 0.001
No	104 (68.4%)	199 (83.6%)	303 (77.7%)	

† Fisher Test was used in variables with frequencies <5.

**Table 3:** Logistic regression model predicting sexual dysfunction and independent variables.

Variable	Case Group		Control Group		Total	
	*p*	OR (CI 95%)	*p*	OR (CI 95%)	*p*	OR (CI 95%)
**Age**	0.009	1.07 (1.01−1.12)	0.386	01.02 (0.97−1.07)	0.159	0.97 (0.94−1.00)
**Hypothyroidism**	-	-	-	-	0.002	2.29 (1.36−3.88)

Only significant results are shown.

**Table 4:** Mean differences (ANCOVA) in sexual dysfunction in case and control groups adjusted for age.

Variables	Case Group (*n* = 152)		Control Group (*n* = 238)		F	*p*
	M (SD)	CI Adjusted	M (SD)	CI Adjusted		
**Desire**	10.03 (0.18)	(9.67−10−40)	11.13 (0.14)	(10.84−11.42)	21.159	*p* < 0.001
**Arousal**	10.93 (0.20)	(10.53−11.34)	12.40 (0.16)	(12.07−12.72)	30.791	*p* < 0.001
**Lubrication**	3.76 (0.07)	(3.61−3.91)	4.29 (0.06)	(4.17−4.41)	28.481	*p* < 0.001
**Orgasm**	3.86 (0.09)	(3.68−4.04)	4.25 (0.07)	(4.10−4.40)	10.673	0.001
**Penetration Pain**	12.07 (0.18)	(11.71−12.44)	13.123 (0.14)	(12.83−13.41)	19.395	*p* < 0.001
**Anticipatory Anxiety**	4.30 (0.07)	(4.16−4.44)	4.57 (0.05)	(4.46−4.68)	8.651	*p* = 0.003
**Sexual Initiative**	2.66 (0.09)	(2.48−2.83)	3.07 (0.07)	(2.93−3.21)	12.639	*p* < 0.001
**Sexual Communication**	3.60 (0.10)	(3.39−3.80)	3.91 (0.08)	(3.75−4.08)	5.586	0.019
SeAcSa *	7.99 (0.15)	(7.68−8.30)	8.75 (0.12)	(8.50−9.00)	14.260	*p* < 0.001
GeSeSa **	3.32 (0.09)	(3.14−3.51)	3.69 (0.07)	(3.54−3.84)	9.158	0.003

* SeAcSa = Sexual Activity Satisfaction; ** GeSeSa = General Sexual Satisfaction.
